# Impact of *OTAbZIP* on Ochratoxin A production, mycelium growth and pathogenicity of *Aspergillus westerdijkiae* under water activity stress

**DOI:** 10.1080/21501203.2024.2355333

**Published:** 2024-06-05

**Authors:** Yanling Ma, Mingxuan Li, Tanvir Ahmad, Yingyao Deng, Muyuan Zhuang, Guangyou Tan, Yang Liu

**Affiliations:** a School of Food Science and Engineering, Foshan University/National Technical Center (Foshan) for Quality Control of Famous and Special Agricultural Products/Guangdong Key Laboratory of Food Intelligent Manufacturing, Foshan, China; b Guangdong Provincial Key Laboratory of Protein Function and Regulation in Agricultural Organisms, College of Life Sciences, South China Agricultural University, Guangzhou, China

**Keywords:** *OTAbZIP*, OTA biosynthesis, water activity, *Aspergillus westerdijkiae fc-1*, pathogenicity

## Abstract

*Aspergillus westerdijkiae* is a major producer of ochratoxin A (OTA), a highly toxic and carcinogenic mycotoxin found in various food and feed products. *A. westerdijkiae* produces excessive amount of OTA under various water activity (a_w_) conditions that occur during food and feed storage. The biosynthetic gene clusters associated with OTA production include *OTAbZIP*, which plays a key role in controlling mycotoxin production in response to environmental conditions. This study explored the regulation of OTA biosynthesis in *A. westerdijkiae fc-1*, focusing on the *OTAbZIP* gene’s influence under a_w_ stress. The mycelium growth of *A. westerdijkiae fc-1* wild type and *OTAbZIP* mutant strains increased by 40.7% and 50.5% under high water activity (0.96 a_w_) respectively, at 6 days post-inoculation (dpi), indicating a stress on *A. westerdijkiae fc-1*. While *OTAbZIP* mutant did not produce OTA under both high and moderate a_W_ conditions. The wild type produced OTA and OTA biosynthetic gene expression levels were downregulated under high (0.96 a_w_) and moderate (0.91 a_w_) water activity. The expression level of *hog1* gene in *OTAbZIP* mutant was significantly lower than in the wild type. Pathogenicity tests revealed that deletion of *OTAbZIP* did not significantly affect disease infection. This study shows that deleting *OTAbZIP* gene greatly reduces OTA production, affecting the strain’s adaptability to water activity stress.

## 1. Introduction

Ochratoxin A (OTA) producing fungi widely contaminate various foods and their byproducts resulting in the substantial production of OTA. OTA causes high levels of toxicity including carcinogenicity, hepatotoxicity, genotoxicity, cytotoxicity, and immunotoxicity. OTA is listed as a 2-B carcinogen by the International Agency for Research on Cancer, which seriously threatens food safety and endangers human health (Gonzalez et al. 2020; Kumar et al. 2020). The National Cancer Institute/National Toxicology Program (NCI/NTP) demonstrated that OTA is the most potent renal carcinogen ever studied in rodents (National Toxicology Program 1989). Several species of *Aspergillus* and *Penicillium* produce OTA, which has been detected in a variety of common cereals such as rice, wheat, corn, barley, and oats, as well as in grapes, spices, coffee, grape juice, and other agricultural products (Zhihong et al. 2015). In addition, OTA has also been found in herbal medicines (Özden and Özden 2018; Zhu et al. 2022), food additives (Solfrizzo et al. 2015), and water (Mata et al. 2015).

The mycelium growth and mycotoxin production of *Aspergillus* species are influenced by various environmental factors, including water activity, pH, light, and temperature. Among these factors, water activity (a_w_) stands out as a key determinant affecting the production of mycotoxins during the storage of food and feed ingredients (Cervini et al. 2020; Mutlu-İNgök et al. 2020; Priesterjahn et al. 2020). Water activity serves as a crucial indicator for assessing the moisture content in food. In general, foods with high a_w_ are more susceptible to fungal infection and spoilage compared to those with low a_w_. OTA-producing fungi *Aspergillus* and *Penicillium* species have the potential to contaminate foods both in high and low a_w_ conditions. The influence of a_w_ extends to the growth and secondary metabolism of fungi, consequently regulating the production of mycotoxins (Kapetanakou et al. 2009; Wang et al. 2018; Zhang et al. 2021). Fungal species possess a signalling pathway known as the HOG-MAPK (high osmolarity glycerol mitogen-activated protein kinase) pathway, which plays a critical role in responding to a diverse range of environmental stresses. These stresses include osmotic, oxidative, a_w_, cold, heat, and light (Fernandes et al. 2015; Bersching and Jacob 2021; Mo et al. 2021). The *hog1* gene in the HOG-MAPK pathway plays a vital regulatory role under a_w_. Knockout of the *afsakA* (*hog1*) gene increases the sensitivity of *Aspergillus flavus* to a_w_ stress and induces toxin production, indicating that *hog1* is involved in the a_w_ stress regulation of growth, sporulation and toxin production in *A. flavus* (Zhang et al. 2014, 2015). Understanding the regulatory mechanism of a_w_ on mycotoxins is beneficial during the storage of grain and feed materials, aiding in the prevention and control of mycotoxin contamination in these substances. Currently, several studies have investigated the biosynthesis pathway of OTA including the biosynthesis mechanism of OTA such as polyketide synthase gene *otaA*, the nonribosomal polypeptide synthase gene *otaB*, the P450 oxidase gene *otaC*, the halogenase gene *otaD*, and the *bZIP* transcription factor gene *otaR1* which are directly implicated in the process of OTA biosynthesis (Wang et al. 2015, 2016). Functioning as a *bZIP* regulator, *otaR1* governs the expression of four biosynthetic genes: *otaA*, *otaB*, *otaC*, and *otaD*. The bZIP protein is a conserved, specific pathway-transcription factor found in eukaryotes, and it regulates a congregation of genes associated with environmental stress adaptation (Hurst 1995; Wang et al. 2018). The *bZIP* transcription factor *atfA* in *Penicillium marneffei* is partially involved in the regulation of oxidative stress but not in the regulation of osmotic and UV light stress (Nimmanee et al. 2014). Knock-out mutants of the *bZIP* transcription factor *fvatfA* in *Fusarium verticillioides* are more sensitive to oxidative stress factors (Szabó et al. 2020). The overexpression of the OTA biosynthesis gene cluster was observed in *A. carbonarius* under conditions of high water activity (a_w_ = 0.99). Notably, the expression level of the *bZIP* gene was found to be the highest, suggesting that *bZIP* plays a crucial role in the regulation of a_w_ stress in *Aspergillus* species (Cervini et al. 2020). A novel *bZIP* transcription factor gene, *atfC*, has been identified in *A. flavus*, demonstrating its advantageous role in enhancing *A. flavus* tolerance under drought stress conditions (Fountain et al. 2020). The mechanism of the *bZIP* response to a_w_ stress in *A. westerdijkiae* remains elusive. To unveil this mechanism, we introduced glycerol into the culture medium to simulate a_w_ stress. This approach aims to elucidate how the *OTAbZIP* gene regulates the mycelium growth and OTA production of *A. westerdijkiae fc-1* under a_w_ stress.

## 2. Materials and methods

### 2.1. Fungal strain and culture mediums

The fungus studied in the current research is OTA producing *A. westerdijkiae fc-1* (accession number PRJNA264608). Potato dextrose agar (PDA) medium and potato dextrose broth (PDB) medium supplemented with glycerol were utilised. The a_w_ of the PDA/PDB medium was maintained at 0.98 a_w_, 0.96 a_w_, 0.95 a_w_, 0.93 a_w_, 0.91 a_w_, and 0.88 a_w_ by adding different amounts (v/v) of glycerol (Nakagawa and Oyama 2019). Both PDA and PDB media were subjected to autoclaving at 121 °C for 20 min to facilitate the morphological characterisation and determination of OTA production in *A. westerdijkiae fc-1*.

### 2.2. *Identification of the* OTAbZIP *gene*

The characteristics of the *OTAbZIP* gene were analysed by conducting a BLASTp alignment, comparing the *OTAbZIP* gene with the bZIP genes found in currently known fungi. This analysis was carried out on the NCBI website (https://www.ncbi.nlm.nih.gov/).

### 2.3. *Knockout of the* OTAbZIP *gene in* A. westerdijkiae fc-1

Primer design was executed using Primer Five-Software. The promoter and terminator regions (−1.5 kb) were subsequently amplified from the *A. westerdijkiae fc-1* genome utilising Top-Taq DNA polymerase from Bioron GmbH, Ludwigshafen, Germany.

The PCR programme is performed according to Table 1. The PCR-amplified DNA, along with 5 μL of sodium heparin, was introduced into *A. westerdijkiae fc-1* protoplasts. Positive transformants were then screened through three passages on PDA supplemented with 100 μg/mL hygromycin B, and homology-adjusted PCR patterns corresponding to T-DNA at the target site were assessed.

**Table 1.** Knockout PCR programme of the *OTAbZIP* gene in *Aspergillus westerdijkiae fc-1.*

Temperature (℃)		Time
	95	10 min
35X 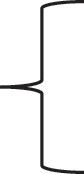	95	30 s
	54	30 s
	72	1 min 30 s
	72	7 min
	4	∞

The *A. westerdijkiaefc-1* protoplasts were solubilised and subjected to centrifugation at 4 °C and 5,000 r/min for 5 min. The resultant filter residue was collected, and 800 μL of STC solution (comprising 0.8 mol/L sorbitol, 50 mmol/L Tris-HCl, and 50 mmol/L calcium chloride) was added to re-suspend the protoplasts. This suspension was then gradually added dropwise to 200 μL of SPTC solution (containing 0.8 mol/L sorbitol, 40% polyethylene glycol, 50 mmol/L Tris-HCl, and 50 mmol/L calcium chloride), thoroughly mixed, and kept on ice for subsequent use.

The PCR cloned DNA solution was transformed, and 5 μL of sodium heparin (0.9 U/L) was mixed gently with the *A. westerdijkiae fc-1* protoplasts and allowed to stand on ice for 30 min, with periodic mixing to prevent the formation of protoplasts. Precipitation affects transformation efficiency. Then, one-third volume of SPTC solution was added dropwise, mixed gently, and allowed to stand at room temperature for 20 min. The protoplasts were then plated on a medium containing hygromycin for selection. After incubating at 28 °C for 3 d, the fungi exhibiting normal growth were transferred to PDB medium without hygromycin and shaken at 28 °C and 180 r/min for 3 d to obtain the transformed cells of *A. westerdijkiae fc-1*.

### 2.4. Phenotypic characterization

The *ΔOTAbZIP* mutant and wild-type strains were inoculated on a PDA medium with varying water activity (0.98 a_w_, 0.96 a_w_, 0.95 a_w_, 0.93 a_w_, 0.91 a_w_, and 0.88 a_w_). The inoculated plates were then kept in the dark at 28 °C, and the colony diameter was observed and measured on the 3 and 6 dpi. The inhibition was calculated using the formula.

$$\mathrm{Fu}\mathrm{ngal}\mathrm{inhibition}\left(\%\right)=\left[\mathrm{Control}-\left(\frac{\mathrm{Sample}}{\mathrm{Control}}\right)\right]\times 100\%$$

### 2.5. Pathogenicity test

The ripe pear (Sydney var.) fruit was detached from the stem, and its surface was subjected to sterilisation three times using 75% alcohol, followed by allowing it to evaporate in ultra clean laminar flow chamber. Subsequently, 10 μL of conidial suspension (10^7^ spores/mL) from both the wild-type strain and mutant *fc-1* strain was injected into the pear fruit using a needle syringe. for control, the spore suspension was replaced with an equivalent volume of sterile water. The inoculated fruits were incubated at 28 °C for 3, 6, and 9 dpi. The growing colony diameter of the infection site on the fruit was measured at 3, 6 and 9 dpi.

### 2.6. OTA detection

The *ΔOTAbZIP* mutant and the wild-type *A. westerdijkiae fc-1* strains were inoculated on PDB medium containing various concentrations of glycerol to maintain varying a_w_ conditions and incubated at 28 °C and 180 r/min. The OTA content was analysed at 6 and 9 dpi. For HPLC sample preparation, 5 mL of culture was mixed with 10 mL of methanol (Sigma-Aldrich Inc., St. Louis, USA) in a 50 mL centrifuge tube. The tube was vigorously shaken using a shaker (Crystal Technology & Industries, Inc. ISRDD3, Suzhou, China) for 3 min and sonicated for 30 min. The samples were then centrifuged at 5,000 r/min for 10 min using a centrifuge (Eppendorf Inc., Hamburg, Germany). The supernatant (1.5 mL) in the centrifuge tube was extracted and filtered through a 0.22 μm organic microporous filter (Bkmam Biotechnology Inc., Hunan, China). The concentration of OTA was detected using HPLC-FLD (Agilent Inc., California, USA) detector. Agilent 1260 analytical liquid chromatography coupled with an FLD-fluorescence detector was used. The excitation and emission wavelength were set at 336 nm and 440 nm respectively. Agilent ZorbaxSB-C18 column (4.6 mm × 250 mm, 5 μm) was used for the detection of OTA and mobile phase run as acetonitrile: 2% acetic acid water (60/40, v/v) with the flow rate of 1.0 mL/min. The sample injected volume was 20 μL and the detection time was set to 10 minutes for each sample.

### 2.7. Detection of gene expression by RT-qPCR

The *ΔOTAbZIP* mutant and the wild type of strain were inoculated in a PDB medium containing different concentrations of glycerol and placed at 28 °C and 180 r/min in the dark. The relative expression levels of the OTA biosynthesis genes *otaA*, *otaB*, *otaC*, *otaD*, and *hog1* were determined at 3 dpi. Primers were designed according to the target gene to be detected. After extracting RNA from the sample, the integrity was checked by electrophoresis, and the purity and concentration were checked by measuring the OD value. One microgram of RNA was synthesised into cDNA using M-MLV reverse transcriptase (Life Technologies, Milan, Italy) and primers for the *bZIP* gene. The expression levels of OTA biosynthetic gene cluster genes (*otaA*, *otaB*, *otaC*, and *otaD*) and *hog1* were assessed using a real-time PCR detection system. The amplification conditions comprised 45 cycles of 95 °C for 3 min, followed by 95 °C for 10 s, and 60 °C for 45 s. The relative gene expression was calculated utilising the CFX Manager software from Bio-Rad Laboratories and the 2^−ΔΔCT^ method, with triplicate measurements for every sample.

## 3. Results

### 3.1. *Identification of the* OTAbZIP *gene*

**Figure 1.**
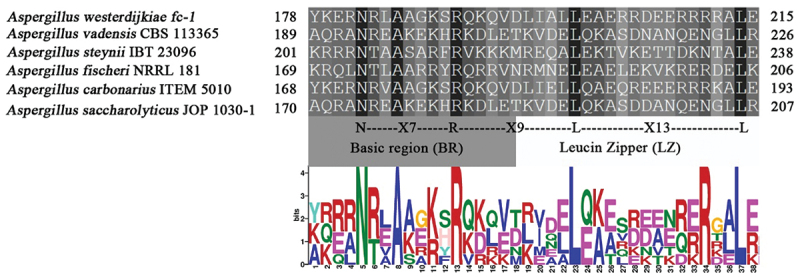
Correlation between the br-LZ domain of the *OTAbZIP* protein and MEME prediction in *Aspergillus westerdijkiae fc-1* and other *Aspergillus* species.

The *OTAbZIP* gene of *A. westerdijkiae fc-1* is located in Scaffold_Scf_8 of the *A. westerdijkiae fc-1* genome, with a length of 767 bp, and it encodes a protein of 263 aa. In *A. fischeri*, *A. vadensis*, *A. carbonarius*, *A. saccharolyticus*, and *A. steynii*, according to the calibration and prediction of the fungal BRLZ domain, the conserved canonical N-X7-R region BR domain, R-X9-L region, distinguishable BR domain and LZ domain and at least four leucine residues of the LZ domain were identified (Figure 1), indicating that the *OTAbZIP* gene is a bZIP-regulated gene.

### 3.2. *Knockout of the* OTAbZIP *gene in* A. westerdijkiae fc-1

**Figure 2.**
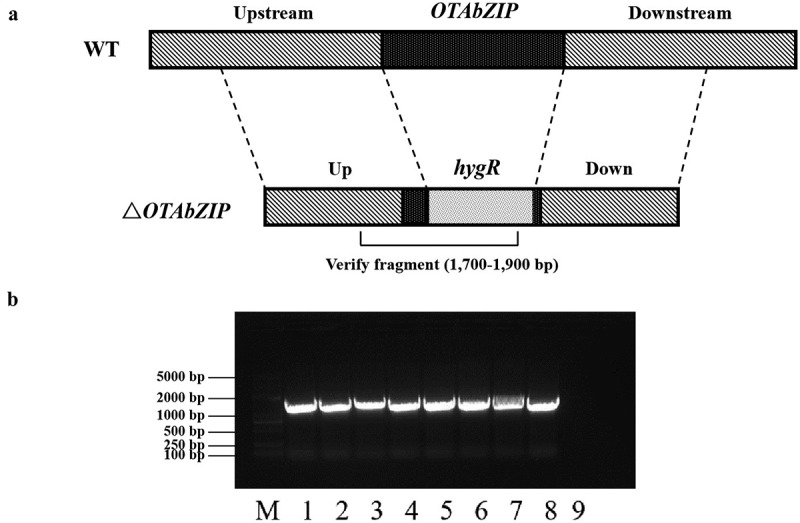
(a) Identification of the hygromycin gene in the *∆OTAbZIP* mutant *Aspergillus westerdijkiae*. (b) Strategy of gene replacement. Agarose gel electrophoresis showing PCR amplified products for wild type and ∆*OTAbZIP* mutant *A. westerdijkiae fc-1*. M: DL 5000 DNA Marker; 1–8: ∆*OTAbZIP* mutant *A. westerdijkiae fc-1*; 9: Wild-type *A. westerdijkiae fc-1*.

To investigate the role of *OTAbZIP* in OTA biosynthesis, this gene was substituted with a hygromycin resistance gene in the *A. westerdijkiae fc-1* strain. Δ*OTAbZIP* mutants were chosen for examination, and the number of T-DNA copies in the genome was determined through qPCR, using the wild-type strain as a control. In the *OTAbZIP* mutant *A. westerdijkiae fc-1* strain, the amplified band was 1,700–1,900 bp, representing a part of the replaced hygromycin gene, indicating that the *∆OTAbZIP* gene was successfully knocked out, and the mutant strain showed a visible band after amplification (Figure 2). On the contrary, there were no target bands in the wild-type *A. westerdijkiae fc-1* strain.

### 3.3. Phenotypic characterization

**Figure 3.**
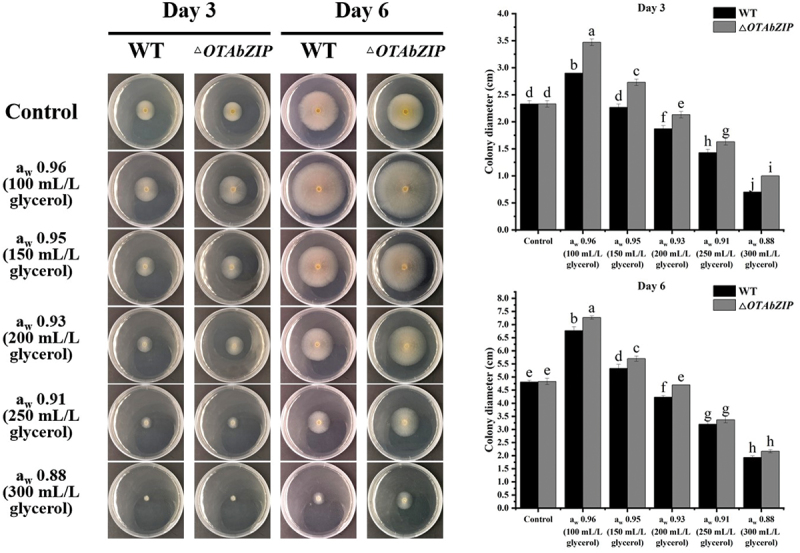
Mycelium growth of wild type and *∆OTAbZIP* mutant *Aspergillus westerdijkiae fc-1* strains in different a_w_ culture medium. WT: Wild-type *A. westerdijkiae fc-1*; ∆*OTAbZIP*: ∆*OTAbZIP* mutant *A. westerdijkiae fc-1*; a_w_: Water activity. Different lowercase letters represent significant differences, *p* < 0.05.

The wild-type strain exhibited enhanced growth at 0.96 a_w_, with increases of 24.4%. Conversely, growth inhibition was observed at 0.95 a_w_, 0.93 a_w_, 0.91 a_w_, and 0.88 a_w_, with inhibition rates of 2.7%, 19.7%, 38.6%, and 59.9%, respectively, at 3dpi. In comparison, the *ΔOTAbZIP* mutant demonstrated improved growth at 0.96 a_w_ and 0.95 a_w_, with increases of 48.9% and 7.2%, respectively. Growth inhibition for the *ΔOTAbZIP* mutant commenced at 0.93 a_w_, 0.91 a_w_, and 0.88 a_w_, with inhibition rates of 8.6%, 30.0%, and 57.1%, respectively. These results suggest that the mutant strains have a higher tolerance to water action. On 6 dpi, the wild-type strain exhibited enhanced growth at 0.96 a_w_ and 0.95 a_w_, with increases of 40.7% and 10.8%, respectively. Conversely, growth inhibition was observed at 0.93 a_w_, 0.91 a_w_, and 0.88 a_w_, with inhibition rates of 12.1%, 33.5%, and 59.9%, respectively. In comparison, the *ΔOTAbZIP* mutant demonstrated improved growth at 0.96 a_w_ and 0.95 a_w_, with increases of 50.5% and 18.4%, respectively. Growth inhibition for the *ΔOTAbZIP* mutant commenced at 0.91 a_w_ and 0.88 a_w_, with inhibition rates of 30.2% and 55.1%, respectively. Importantly, the inhibition rates were lower than those observed in the wild type (Figure 3). These findings indicate increased tolerance to water activity in the *ΔOTAbZIP* mutant strains.

### 3.4. Pathogenicity test

**Figure 4.**
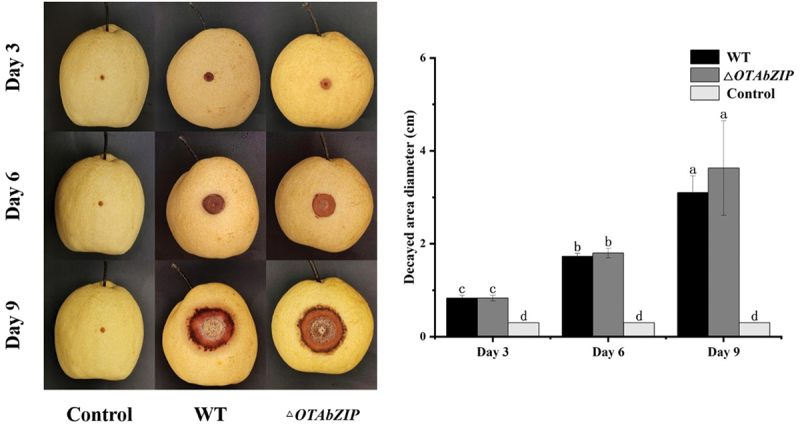
Disease infection of wild type and ∆*OTAbZIP* mutant *Aspergillus westerdijkiae fc-1* strains on pear fruit. WT: Wild-type *A. westerdijkiae fc-1*; ∆*OTAbZIP*: ∆*OTAbZIP* mutant *A. westerdijkiae fc-1*. Different lowercase letters represent significant differences, *p* < 0.05.

The disease infection of pear fruit showed scabs caused by both wild-type and mutant *A. westerdijkiae fc-1* at 3, 6, and 9 dpi (Figure 4). The size of the decayed area was not significantly different in either wild type or mutant strains, indicating that the pathogenicity of the mutant strain with the *OTAbZIP* gene was not significantly different from that of the wild type. The deletion of *OTAbZIP* does not affect the disease infection.

### 3.5. OTA detection

**Figure 5.**
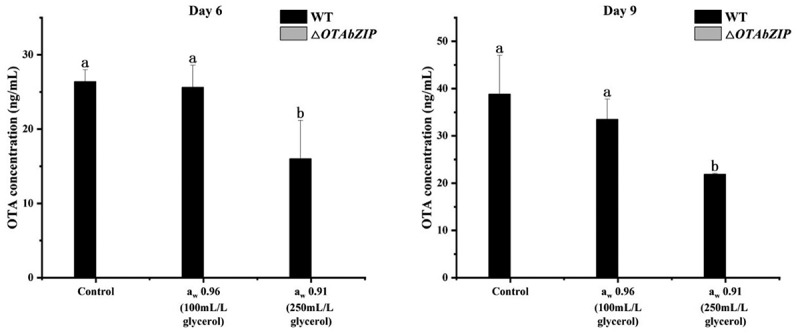
OTA production of the wild type and ∆*OTAbZIP* mutant *Aspergillus westerdijkiae fc-1* strains under different a_w_ conditions. WT: Wild-type *A. westerdijkiae fc-1*; ∆*OTAbZIP*: ∆*OTAbZIP* mutant *A. westerdijkiae fc-1*; a_w_: Water activity. Different lowercase letters represent significant differences, *p* < 0.05.

The wild-type *A. westerdijkiae fc-1* produces OTA as it is growing. On the 9 days of growth under high water activity conditions (0.96 a_w_), the toxigenicity of the wild-type strain was inhibited by 13.7% compared to the control. Similarly, under conditions of moderate water activity (0.91 a_w_), the toxigenicity of the wild-type strain was inhibited, with a decrease of 43.6%. These results suggest that both high a_w_ and moderate a_w_ are not conducive to the toxigenic production of *A. westerdijkiae fc-1*. Additionally, the toxigenic production of *A. westerdijkiae* is more sensitive to the addition of glycerol and changes in a_w_. However, OTA was not detected in the *∆OTAbZIP* mutant strain on the 9 days of growth (Figure 5), indicating that the strain lost its toxigenic ability following the deletion of *OTAbZIP*.

### 3.6. Detection of gene expression by RT-qPCR

**Figure 6.**
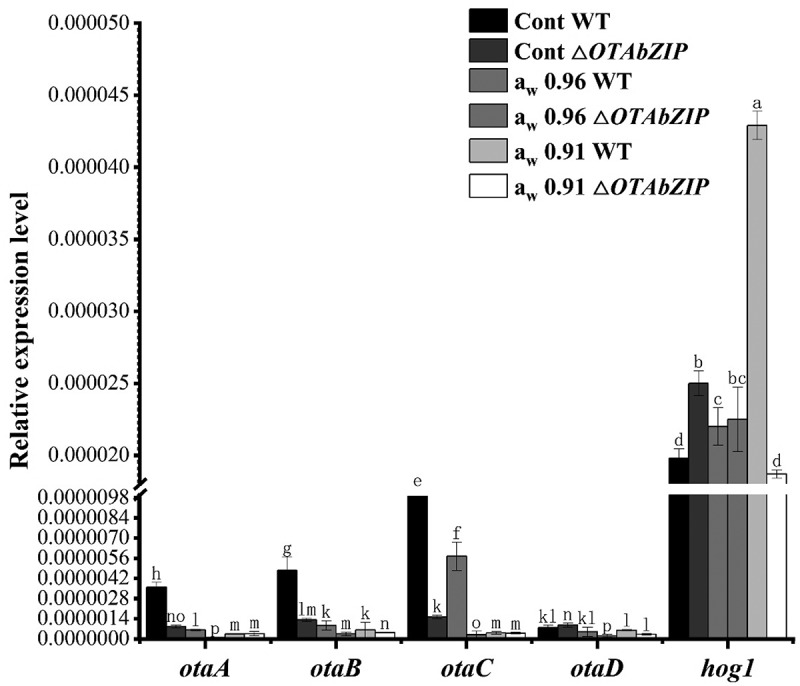
OTA biosynthesis gene (*otaA*, *otaB*, *otaC*, and *otaD*) and *hog1* gene expression of the wild type (WT) and ∆*OTAbZIP* mutant *Aspergillus westerdijkiae fc-1* strains under different aw treatment. Cont: Control group, 0 mL/L glycerine; a_w_ 0.96: 100 mL/L glycerine; a_w_ 0.91: 250 mL/L glycerine. Different lowercase letters represent significant differences, *p* < 0.05.

To investigate the regulatory function of the transcription factor *OTAbZIP* on the expression of OTA biosynthesis genes, the expression levels of OTA biosynthesis genes (*otaA, otaB, otaC*, and *otaD*) were analysed in both wild-type and mutant strains under different water activity conditions (0.96 a_w_ and 0.91 a_w_). Expression levels were analysed (Figure 6) and under high water activity (0.96 a_w_) conditions, the wild type of OTA biosynthesis genes was downregulated compared with the control group (decrease in *otaA*, 82.9%; *otaB*, 80.9%; *otaC*, 41.8%; and *otaD*, 36.4%), and the OTA biosynthesis genes of the mutant strain were downregulated (decrease in *otaA*, 88.8%; *otaB*, 73.8%; *otaC*, 80.1%; and *otaD*, 77.9%). Under moderate water activity, (0.91 a_w_), the wild type of OTA biosynthesis genes was basically downregulated (decrease in *otaA*, 91.4%; *otaB*, 87.2%; *otaC*, 95.9%; and *otaD*, 22.0%), and the OTA biosynthesis genes of the mutant strain were downregulated (decrease in *otaA*, 57.6%; *otaB*, 66.2%; *otaC*, 74.8%; and *otaD*, 68.4%). The results show that the expression of OTA biosynthesis genes in wild-type and mutant *A. westerdijkiae* can be inhibited under both high-water activity and moderate water activity conditions. The lower the water activity was the greater the decrease in the expression of OTA biosynthesis genes in the wild type of strain (the opposite occurred for the mutant strain). For the core gene *hog1* in the HOG-MAPK pathway, under high water activity (0.96 a_w_), the wild-type *hog1* gene was upregulated by 10.5%, and the mutant *hog1* gene was downregulated by 9.6% (no significant difference); under moderate water activity (0.91 a_w_), the wild-type *hog1* gene was upregulated by 121.1%, and the mutant *hog1* gene was downregulated by 25.3%. The findings indicate that the expression of the *hog1* gene in wild-type *A. westerdijkiae* was upregulated under both high and moderate water activity conditions, with a more pronounced upregulation as water activity decreased. On the other hand, in the mutant *A. westerdijkiae*, *hog1* gene expression was downregulated under both high and moderate water activity scenarios, with a more significant downregulation becoming more pronounced as water activity dropped.

## 4. Discussion

In majority of *Aspergillus* species *A. fischeri* NRRL 181, 66.67% homology (Mead et al. 2019); *A. vadensis* CBS 113365, 80.90% homology (de Vries et al. 2005); *A. carbonarius* ITEM 5010, 71.15% homology (Gerin et al. 2021); *A. saccharolyticus* JOP 1030-1, 66.18% homology (Yang et al. 2016); and *A. steynii* IBT 23096, 71.07% homology (Gil-Serna et al. 2015) the *bZIP* gene is located in the OTA biosynthetic gene cluster, with a length of 767 bp, encoding a 263 aa protein. bZIP is a class of transcription factors ubiquitous in plants, animals and microorganisms and consists of two parts: the basic region (BR) and the leucine zipper (LZ) region. Based on homologous sequence comparison analysis (Figure 1), the conserved structural domains of bZIP in *A. westerdijkiae* were identified and the BR and LZ domains could be distinguished, suggesting that OTAbZIP is a bZIP transcriptional regulator.

In the glycerol-conditioned system, higher water activity favoured *A. westerdijkiae fc-1* growth, while as glycerol continued to increase, water activity decreased, leading to inhibition of *A. westerdijkiae fc-1* growth.

The results of the growth experiment of *A. westerdijkiae fc-1* showed that high water activity (0.96 a_w_) promoted the growth of *A. westerdijkiae fc-1* strains, indicating that glycerol was necessary for the growth of *A. westerdijkiae fc-1*, and this glycerol concentration was beneficial to the growth of *A. westerdijkiae fc-1*; moderate water activity conditions of 0.91 a_w_ inhibited the growth of *A. westerdijkiae fc-1* strains, indicating that although glycerol is necessary for the growth of *A. westerdijkiae fc-1*, when the concentration is too high, it has a stress effect on *A. westerdijkiae fc-1*, which is not conducive to the growth of *A. westerdijkiae fc-1*. Compared with the wild type, the mutant strain grew better under the condition of high-water activity (0.96 a_w_) and moderate water activity (0.91 a_w_), the growth of the mutant strain was still better than that of the wild type, indicating that the mutant strain was more tolerant to water activity than the wild type, and the *bZIP* gene increased the strain’s sensitivity to water activity. Among other toxin-producing fungi, there are cases where their growth is regulated by water activity, the optimal growing conditions of A. *flavus* are 0.99 a_w_ and 30 °C, and its growth rate is regulated by the water activity of the growth medium and decreases with decreasing water activity (Romero Donato et al. 2022). At 0.99 a_w_, *A. westerdijkiae fc-1* grows at the highest rate, moreover, the *bZIP* gene expression in its OTA synthesis gene cluster is the highest at this water activity (Cervini et al. 2020). The above results indicate that water activity has a significant regulatory effect on the growth of toxin-producing fungi, with high water activity (0.96 a_w_) usually promoting growth and medium water activity (0.91 a_w_) beginning to inhibit growth.

Generally, bZIP can regulate the pathogenic fungi’s virulence by affecting its mycotoxins production and growth (Leiter et al. 2021). The bZIP-type transcription factor of *F. verticillioides*, FvAtfA, its knockout mutant strain is less virulent in tomatoes than in WT (Szabó et al. 2020). But the results of the pathogenicity test of *A. westerdijkiae fc-1* showed that the wounds infected with Sydney pears treated with the spore suspensions of the wild type and mutant strains had scabs and spores, and the size of the rotten area was basically the same between the wild type and mutant strains, indicating that the *OTAbZIP* gene had no obvious influence on the pathogenicity of *A. westerdijkiae fc-1*.

The results from the toxicity experiment of *A. westerdijkiae fc-1* revealed a decrease in toxin production for the wild-type strain under high water activity (0.96 a_w_) and a further decreased under moderate water activity (0.91 a_w_). This suggests that the toxin production of *A. westerdijkiae fc-1* is influenced by the degree of water activity. Notably, the mutant strains did not produce any toxicity under any water activity conditions. Upon combining the results of the biosynthetic gene expression analysis, it was observed that the expression levels of the OTA biosynthetic genes (*otaA*, *otaB*, *otaC*, and *otaD*) were inhibited under conditions of high-water activity (0.96 a_w_) in the wild-type *A. westerdijkiae fc-1*. Under moderate water activity (0.91 a_w_) conditions, the expression levels of these biosynthetic genes in the mutant strain were inhibited to extremely low levels. Additionally, the expression levels of the OTA biosynthetic genes in the wild-type strain were also inhibited, with *otaD* showing an opposite trend.

This result is consistent with the results of the toxin production experiment. At 0.95 a_w_, *F. graminearum* produced the largest and fastest amount of deoxynivalenol (DON) (Ramírez Albuquerque et al. 2022). *Alternaria arborescens* produces the most toxins at 0.995 a_w_ and decreases as a_w_ decreases (Vaquera et al. 2014). Likewise, the deletion of the *OTAbZIP* gene greatly reduced the expression level of the OTA biosynthesis genes, resulting in the loss of the toxigenic ability of *A. westerdijkiae fc-1*, indicating that *OTAbZIP* controls the production of OTA by regulating the expression of OTA biosynthesis genes.

*Hog1* is a component of the HOG-MAPK pathway that is closely related to the water activity stress signal (Fernandes et al. 2015). The experimental results showed that the reduction in water activity in the wild-type of strain increased the expression level of the *hog1* gene, while the mutant strain showed the opposite effect. Decreased water activity decreased the expression level of the *hog1* gene. This indicates that the *OTAbZIP* gene is indeed involved in the expression of OTA biosynthesis genes and that OTA biosynthesis is mediated by water activity. In wild-type strains, with the increase in glycerol concentration, water activity decreased, resulting in drought stress and activation of the HOG-MAPK pathway, promoting the expression of the *hog1* gene in the wild type, which greatly alleviated the damage of the stress to the strain. After knocking out *OTAbZIP*, the water activity that stimulated the HOG-MAPK pathway was cut off, the HOG-MAPK pathway was not activated, and the water activity was reduced (Zhang et al. 2015; Mo et al. 2021). The reduction damaged the strain, and then the growth of the strain and the expression of *hog1* were inhibited, so the deletion of the *OTAbZIP* gene greatly reduced the adaptability of the strain to water activity stress.

## Supplementary Material

Supplemental Material
