# Supplementary material for: Impact of OTAbZIP on Ochratoxin A production, mycelium growth and pathogenicity of Aspergillus westerdijkiae under water activity stress
Source: Mycology. 2024 Jun 5;15(4):620–30. doi: 10.1080/21501203.2024.2355333 (PMC11636139; doi:10.1080/21501203.2024.2355333)
Supplement: Supplemental Material [file TMYC_A_2355333_SM4029.docx]

| Primer | Sequence (5’ to 3’) |
| --- | --- |
| *OTAbZIP*-Up-F | CCGAGCGGTCAAGATTCCATA |
| *OTAbZIP*-Up-R | CAAAATAGGCATTGATGTGTTGACCTCCACGCTGTCATCAAAAGGCAGA |
| *OTAbZIP*-Down-F | CTCGTCCGAGGGCAAAGGAATAGAGTAGGGGCTTCTAGCACAGGACAT |
| *OTAbZIP*-Down-R | CGACGCAATGTGACCTACGA |
| *OTAbZIP*-Knock-F | CAGGTCGGCGCTTCTTATCT |
| *OTAbZIP*-Knock-R | ATATTGCGTGCGCTTTCTCG |
| *OTAbZIP*-Out-F (Verification) | GTCGTTGATGTGTACTTCC |
| *OTAbZIP*-Out-F (Verification) | TCGTTCACTTACCTTGCTT |

**Primers involved in mutant construction and verification**
